# A Physical Activity Just-in-time Adaptive Intervention Designed in Partnership With a Predominantly Black Community: Virtual, Community-Based Participatory Design Approach

**DOI:** 10.2196/33087

**Published:** 2022-03-28

**Authors:** Maria Cielito Robles, Mark W Newman, Aalap Doshi, Sarah Bailey, Linde Huang, Soo Ji Choi, Chris Kurien, Beza Merid, Joan Cowdery, Jessica R Golbus, Christopher Huang, Michael P Dorsch, Brahmajee Nallamothu, Lesli E Skolarus

**Affiliations:** 1 Department of Neurology University of Michigan Ann Arbor, MI United States; 2 School of Information University of Michigan Ann Arbor, MI United States; 3 Michigan Institute for Clinical & Health Research University of Michigan Ann Arbor, MI United States; 4 Bridges into the Future Flint, MI United States; 5 Department of Internal Medicine University of Michigan Ann Arbor, MI United States; 6 School of Health Promotion and Human Performance Eastern Michigan University Ypsilanti, MI United States; 7 College of Pharmacy University of Michigan Ann Arbor, MI United States

**Keywords:** just-in-time adaptive intervention, design, community participatory design, health equity, hypertension, healthy lifestyle, blood pressure, physical activity

## Abstract

**Background:**

Black people are disproportionally impacted by hypertension. New approaches for encouraging healthy lifestyles are needed to reduce hypertension and promote health equity in Black communities.

**Objective:**

In this report, we describe the early-stage, virtual design of a just-in-time adaptive intervention (JITAI) to increase physical activity in partnership with members of a low-income, predominantly Black community.

**Methods:**

The hallmark of JITAIs is highly contextualized mobile app push notifications. Thus, understanding participants' context and determinants of physical activity are critical. During the height of the COVID-19 pandemic, we conducted virtual discovery interviews and analysis guided by the Behavior Change Wheel (which focuses on participants' capacity, opportunity, and motivation to engage in physical activity), as well as empathy mapping. We then formed a community-academic participatory design team that partnered in the design sprint, storyboarding, and paper prototyping.

**Results:**

For this study, 5 community members participated in the discovery interviews, 12 stakeholders participated in the empathy mapping, 3 community members represented the community on the design team, and 10 community members provided storyboard or paper prototyping feedback. Only one community member had used videoconferencing prior to partnering with the academic team, and none had design experience. A set of 5 community-academic partner design principles were created: (1) keep users front and center, (2) tailor to the individual, (3) draw on existing motivation, (4) make physical activity feel approachable, and (5) make data collection transparent yet unobtrusive. To address community-specific barriers, the community-academic design team decided that mobile app push notifications will be tailored to participants’ baseline mobility level and community resources (eg, local parks and events). Push notifications will also be tailored based on the day (weekday versus weekend), time of day, and weather. Motivation will be enhanced via adaptive goal setting with supportive feedback and social support via community-generated notifications.

**Conclusions:**

We completed early-stage virtual design of a JITAI in partnership with community participants and a community design team with limited design and videoconferencing experience. We found that designing JITAIs with the community enables these interventions to address community-specific needs, which may lead to a more meaningful impact on users' health.

## Introduction

Hypertension is the most important modifiable risk factor for cardiovascular disease, the leading cause of mortality in the United States [1,2]. Black Americans have the highest prevalence of hypertension of any racial/ethnic group in the United States, contributing to their disproportionately high burden of cardiovascular disease [3]. Lifestyle modifications, such as increasing physical activity, play an essential role in hypertension management [4,5]. However, about half of Americans do not meet the American Heart Association recommendation of 150 minutes/week of moderate-intensity physical activity [6], and Black Americans engage in less physical activity than White Americans [6-9]. To improve health equity, new approaches to promote healthy lifestyles are needed.

One approach may be just-in-time adaptive interventions (JITAIs, pronounced as “jedis”) [10-12]. This novel approach incorporates real-time data streams from wearable devices (eg, Apple Watch, Fitbit) to generate tailored notifications delivered at moments when there is a higher likelihood of their successful adoption. Not surprisingly, JITAIs introduce several design considerations, including the timing, frequency, modality, and presentation of intervention support, along with an understanding of the impact of behavior change strategies such as “provide feedback on performance” and “prompt specific goal setting” [13]. In the myBPmyLife project, as part of the Wearables In Reducing risk and Enhancing Daily Lifestyle (WIRED-L) center**,** we are designing and adapting a JITAI to increase physical activity and reduce blood pressure that will then be tested in a randomized controlled trial [11].

The myBPmyLife JITAI is based on a JITAI designed and tested among a predominantly White population [11]. To ensure that JITAI design is inclusive of a low-income, predominantly Black community, we undertook a community-based participatory design approach [14,15]. This paper describes our virtual, early-stage, community participatory approach, as necessitated by the COVID-19 pandemic, grounded in health behavior theory, to adapt a physical activity JITAI to be community-inclusive.

## Methods

### Community-Based Participatory Design Approach

Flint, Michigan, has a population of 95,358 people: 54% are Black people, and 12% of adults have a bachelor’s degree or higher [16]. About 40% of Flint residents self-report hypertension [17]. Furthermore, Flint is recovering from a lead crisis, which may be associated with the development of hypertension [18,19].

Our work in Flint is based on an established community-academic partnership focused on addressing racial disparities in cardiovascular disease, which has been in place for over a decade [20]. The principal community-partner organization is Bridges into the Future, a grassroots community organization dedicated to the health and well-being of the Flint community. The principal academic partner organization is the University of Michigan-Ann Arbor, with support from the University of Michigan-Flint. Before starting the design phase, the myBPmyLife project underwent review and received approval from the Flint Community Ethics Review Board (HUM00181363) [21]. Bridges into the Future led recruitment for discovery interviews and empathy mapping. This involved identifying possible participants with a smartphone or computer access, a willingness to use videoconferencing, and initial interest. Contact information was then provided to the academic team, who obtained consent and delivered videoconferencing training. The community design team was composed of the director of Bridges into the Future (SB) as well as 2 other community members recruited by Bridges into the Future to provide a wider view of the community. The director of Bridges into the Future (SB) and one of the academic partners (LES) led the community advisory board, which consists of representatives from the Flint medical community, including Hamilton Health Center, the largest Federally Qualified Health Center in Flint, and the community-based organization partners. The community partners and community advisory board will continue to partner in designing the app and trial and will lead community dissemination of the results.

### Design Process Overview and Formation of Community Design Team

**Figure 1:**
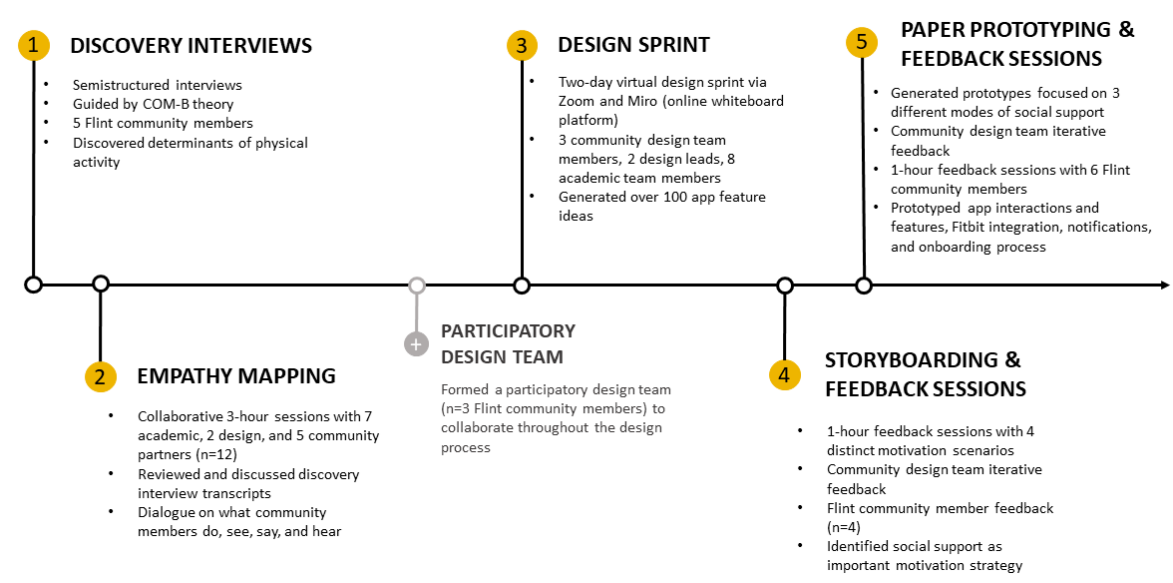
Discovery and design. COM-B: Capability, Opportunity, and Motivation.

Our early-stage design consisted of five components: (1) discovery interviews, (2) empathy mapping, (3) design sprint, (4) storyboarding, and (5) paper prototyping (Figure 1). Only one of the community participants had used videoconferencing prior to the discovery interviews or empathy mapping. After completion of empathy mapping, the academic and community partners determined that technology was a barrier to creativity and innovation. Thus, we formed a community participatory design team, whose members underwent more intensive training on the use of Zoom, a videoconferencing platform, and intensive training with Miro, a virtual collaborative whiteboard platform. Miro and Zoom were selected based on their ease of use and compatibility with phones. We then moved to design sprints, storyboarding, and paper prototyping. The community design team consisted of three Black women aged 32-72 years who provided near real-time, longitudinal feedback.

### Discovery Interviews

We conducted virtual semistructured interviews with Flint adults with hypertension from July to August 2020. The community partners identified individuals interested in the study. The academic team then consented eligible participants and conducted an individualized training session on using the Health Insurance Portability and Accountability Act–approved videoconferencing service Zoom. Our interview guide was based on the Capability, Opportunity, and Motivation (COM-B) model. The Behavior Change Wheel (BCW), which synthesizes 19 established frameworks of behavior change, posits that for individuals’ behaviors to change, they must have the capability, opportunity, and motivation to enact the behavior. The findings from the COM-B–guided interviews were then mapped to the BCW framework to identify candidate behavior change strategies. We also queried participants about smartphone and wearable device usage. All interviews were conducted via Zoom by a primary interviewer and were recorded and transcribed. For analysis, we used a direct content analysis with coding into the COM-B framework [22].

### Empathy Mapping

A collaborative 3-hour empathy mapping session was held with academic partners, designers, and community members who participated in the discovery interviews to contextualize the discovery interviews and gain a collective understanding of end user needs. Empathy mapping allows researchers and designers to hear and understand the end user's struggles and frustrations firsthand and in their own words [23]. Before the session, participants were assigned two transcripts to review, each from the discovery interviews. During the videoconferencing session, participants were asked a series of questions and were instructed to reflect on their perceptions and understanding of the reviewed transcripts. The questions included the following:

1. What does the interview participant think and feel?
2. What does the interview participant see?
3. What does the interview participant hear?
4. What does the interview participant do?
5. What does the interview participant say?

These data were added to the qualitative coding results to enable a deeper understanding of the data.

### Design Sprint

Design sprints are typically conducted in person to move from empathy generation to the intervention design phase [24]. The design sprint aimed to generate as many diverse and creative ideas as possible for a physical activity intervention in the Flint community. Unlike traditional in-person design sprints, we used videoconferencing with Zoom and Miro. Before the first session, a 60- to 90-minute individual training session was conducted with community participants, often with in-home support from a family member or friend on using Zoom and Miro. Facilitators provided additional real-time tech support during the design sessions. Despite these efforts, real-time workarounds, including email and text, were still needed during the design sprint.

The first day of the design sprint was dedicated to the following: (1) identifying the primary challenges to physical activity and (2) developing problem-solving statements. After a short icebreaker, talks were delivered by a digital technology expert (on mobile technology), a physical activity behavior change expert, a research assistant (on the process of data collection), and a leader from the Flint community. Participants were then asked to create “How Might We” statements to launch brainstorming sessions.

The second day was focused on brainstorming and discussing mobile app features that would address the identified challenges. It began with a Lightning Demos activity [24]. Lightning Demos are a preliminary design exercise by which individuals identify and present 2-3 product and service inspirations prior to concept sketching. For the concept sketching portion of the design sprint, we elected to use three exercises: (1) Notes, (2) Ideas, and (3) Crazy Eights. For exercise 1 (Notes), the primary goal was to note concepts and problems we previously discussed and established. During exercise 2 (Ideas), participants were given 15 minutes to elaborate on the ideas. Participants were encouraged to creatively doodle, write sample text headlines, and sketch diagrams, cartoons, and stick figures. The messaging provided was “the crazier the idea, the better!” For the final portion of the brainstorming activity, we used the Crazy Eights exercise, asking participants to create 8 distinct ideas in 8 minutes [24]. Participants then synthesized their ideas into one final concept, which they shared with the group. We discussed the concepts as a group and then voted on the ones we thought would form the core intervention. Following the design sprint, the design team categorized all sketches and notes using Evernote, a digital note organizing tool.

### Storyboarding and Paper Prototyping

The design team used the information generated during the sprint to create storyboards. These storyboards were presented iteratively to our community design team and then during 4 videoconferencing feedback sessions with Flint community members. Community participant identification, recruitment, and videoconferencing training mirrored that of the discovery interviews. During each session, community design team members were shown the 4 storyboards sequentially. Participants were asked for their reactions—specifically, which parts of the storyboard resonated for them and which elements warranted change or were missing. Design team members were present for all sessions, which were conducted virtually and recorded. After each storyboard was discussed, participants were asked to rank them based on the likelihood of use. The highest-rated storyboards guided the design and development of the paper prototypes. A critical insight from storyboard feedback was the importance of social interaction, which we then represented in different forms through paper prototypes of the JITAI. Feedback on the paper prototypes was gathered over videoconferencing iteratively from the community design team and then from 6 community participants.

## Results

Our findings are categorized into two main domains: (1) user needs discovery and (2) design.

### User Needs Discovery

We conducted 5 discovery interviews and 14 stakeholders (7 academic partners, 5 community members, 2 design team members) participated in an empathy mapping session to further contextualize these findings. Guided by the direct content coding using the COM-B framework and enhanced by empathy mapping, we identified unique community physical activity determinants. Physical capability, often due to more chronic physical impairments, was a barrier to physical activity, as was physical opportunity (eg, access to places for older adults to exercise, gym membership costs, and personal safety). Participants reported a desire to exercise more (reflective motivation). On the other hand, automatic motivation (ie, impulses) was a challenge, with community members lamenting how challenging it can be to adhere to a physical activity plan and feeling discouraged by their failure to adhere. These findings were then linked to the BCW to identify behavior change strategies to address the identified barriers and enhance facilitators (Table 1).

**Table 1:** Results from discovery interviews and empathy mapping mapped to behavior change techniques through the Behavior Change Wheel.

Theme	Exemplar quotes	Selected intervention functions	Behavior change technique
Physical capability	*It limits me, very much so, because sometimes I have to walk with a cane with these issues. There's been time when I've fallen, and because I haven't had any physical therapy, I've not been able to get out and do things, my body sort of feels tightened and locked up* [Participant 6] *I can’t run. I can still walk. My knees are not good like they used to be* [Participant 1]	Training Enablement	Instruction on how to perform a behavior (level of physical activity); graded tasks Action planning; social support
Psychological capability	*I think you should at least spend 40 minutes to an hour every day, doing something. Every day. Even if you have small things around the house, or like a 10-minute exercise, just something, some type of physical activity every day, I think, is a must.* [Participant 4]	Enablement	Social support
Social opportunity	*Not a lot of physical activity unless we’re cleaning the house or something like that because everyone will work in the yard in our household but not a lot.* [Participant 2] *My daughter wanted to take me [to the track] but she doesn’t go too much herself* [Participant 3]	Enablement	Social support
Physical opportunity	*The neighborhood has parks but I don’t see anything for seniors. The only things would be the old forest park where you could walk around the park. Other than the rest of the park is surrounded by kids or teenagers. They got basketball courts and play structures for the toddlers but they don’t have tracks for the seniors so it’s not senior friendly in the neighborhood I live in.* [Participant 1] *I would love to be able to have a gym membership. But there's only a certain amount of income—I'm on a fixed income. If I was able to afford it, I certainly would love to be able to…But everything is so expensive, it's difficult*. [Participant 6]	Environmental restructuring Enablement	Prompts/cues; restructure physical environment (or make aware of options) Goal setting
Reflective motivation	*I believe that it is very important. For one, as you get older, things kind of lock up. You want to keep those things loose* [Participant 4]	Persuasion	Credible source
Automatic motivation	*It was easy for me then because I was slim but then I got my stroke and my balance and I couldn’t do things and I was sitting home all the time and gained 100 pounds ever since and have struggled ever since* [Participant 3] *I had a good regimen going and I was doing really good. Then I had to go to the nursing home and hospice more. Then my son went to the rehab center. I wore myself down to the point where it was easier to come home, sit in the couch, and watch NBC and just be snacking*. [Participant 2]	Persuasion Incentivization Environmental restructuring Enablement	Credible source Audit and feedback Prompts/cues Goal setting, self-talk

Participants primarily used their smartphones for email and text messages, followed by scheduling, receiving news and weather information, taking and storing photos, and using social media (eg, Facebook). None of the participants reported using wearable technology.

### Design

The design sprint was facilitated by two members of the design team and attended by 8 academic team members, 2 design team members, and the 3 community design team members. The design exercises yielded approximately 100 app features. Each feature was analyzed as an artifact and tagged into a categorical theme by the design team. Overall, 204 total tags were categorized into 55 themes. The most common tags were motivation (n=22), options (n=13), personalization (n=13), social connection (n=13), and social motivation (n=13). Five design principles emerged from the design sprint and these guided the remainder of the design process (Textbox 1).

**Design principles generated by the community-academic partnership.**

1. Keep the user front and center (users first, then researchers, then technologists).
2. Tailor to the individual (customize features to individual users depending on physical and environmental barriers, as well as personal preferences).
3. Draw on existing motivation (connect activities with the user’s existing motivation, such as social connections or personal goals).
4. Make physical activity feel approachable (broaden the definition of “exercise” or “physical activity” to simply “moving one’s body” in daily life).
5. Make data collection transparent yet unobtrusive for the user (make it clear to the user what data are being collected, and ensure the data are gathered in a minimally intrusive manner).

Based on our user needs discovery and design sprint results, we focused on motivation. Motivational strategies including gamification, social interaction, education, and emotional support were incorporated into storyboards. Overall, the idea of social interaction as part of their physical activity journey most resonated with the community design team and community participants. The concept of social interaction was then broken down into 3 main concepts for paper prototypes. The first prototype was a 1:1 interaction with an accountability buddy. The second prototype focused on developing teams of people who would challenge each other. The final prototype was community-focused and included participant-facing community statistics and community-gathered suggestions. There were mixed opinions on the “accountability buddy” feature, with one participant asking “what if you don’t have anybody?” Two participants noted it might be challenging to create a group of people who have similar availabilities. Lastly, participants viewed the community-focused feature positively, although there was concern about the interpretation of statistics. All were in favor of community-gathered suggestions.

### Design Implications

Tailored notifications are the foundation of JITAIs. Our first major design decision was to tailor push notifications to participants’ respective communities (Table 2). Second, we identified wide variation in physical capability, necessitating tailoring to a participant’s functional capacity. For example, participants with joint pain will be encouraged to engage in shorter, more frequent bursts of physical activity. One behavior change technique to increase automatic motivation is prompts/cues. However, the prompts/cues must be contextualized and, as such, we will tailor push notifications based on day (weekday versus weekend), time of day, and weather. We will enhance reflective and automatic motivation as well as social opportunity and psychological capability by creating a bank of community-generated push notifications to promote social support and app engagement. Community-generated notifications will infuse community voices into the intervention, leading to enhanced authenticity and relatability. Finally, we will acknowledge and support participants when they do not meet their goals through support and adaptive goal setting of steps [25]. Participants will be assigned a weekly task to review their progress over the previous 7 days and select a daily step goal for the upcoming week. The system will automatically suggest a step goal and allow the user to modify the proposed goal within a specified range before accepting it. The notifications will be celebrative when participants reach their goals and support them when they do not reach their goals [26]. During the trial, all participants will be given a wearable. To assess engagement with the push notifications, we will assess the change in step count in the 60 minutes following the push notification. In the intervention, participants will receive daily messages across 4 time periods.

**Table 2:** Results of community-based participatory design approach to just-in-time adaptive interventions.

Overall findings from community design	Just-in-time adaptive intervention design implications
Physical opportunity is a barrier	Tailor push notifications based on enrollment community
Expanding definition of physical activity (eg, household chores, walking up/down stairs)	Include expanded definition in materials and notifications
Limited physical capability/mobility (eg, pain or multiple comorbidities)	Tailor push notifications to participants’ functional capacity
Social support (ie, strong familial and community ties) is a facilitator of physical activity	Community-generated supportive push notifications
High reflective motivation (want to exercise more, but it is hard)	Community-generated supportive push notifications
Automatic motivation is a barrier to physical activity	Contextual tailoring of push notifications based on time, weather, and step count; weekly adaptive step goals; audit and feedback of steps (daily, weekly, monthly)

## Discussion

Partnering with community members who had limited experience with videoconferencing, we completed virtual ideation, design sprint, and early prototyping. Virtual community-based participatory design was facilitated by the creation of a community design team. Our findings highlight that communities have unique needs. Designs that focus on addressing these community-specific barriers and facilitators may increase the efficacy of the JITAIs and ultimately reduce cardiovascular disease disparities.

We initially planned to include the voices of many community stakeholders to understand the breadth of the community experience and capture community innovation. However, the COVID-19 pandemic posed recruitment challenges, as many of our prepandemic recruitment strategies were predicated on in-person interactions with community partners, and there were technological challenges given the lack of videoconferencing experience among nearly all of our participants. After conducting ideation activities over Zoom, we realized that technology was a barrier to creativity. Thus, we created a 2-stage approach to the early design. We initially obtained iterative, near real-time feedback from the community design team, made adaptations, and then shared later prototypes with community users. We found that this 2-stage approach engendered feelings of responsibility among the community design team, increased trust between the community and design team, and optimized the time spent with community participants.

Our community-based participatory design process had several limitations. Our community design team did not include any men, and men were underrepresented among our community participants more generally. Although a community-based participatory research approach to recruitment was successful, other best practices for recruitment, such as face-to-face interaction, were unable to be initiated due to the COVID-19 pandemic [27-29]. Future studies should use strategies that promote the recruitment of men, such as prioritization of men by the study team, including men as part of the recruitment team, and expanding community partners. Of note, a health emergency prohibited our lead male community partner from participating, although women predominantly lead community-based organizations in Flint. Second, we conducted this early phase design during the COVID-19 pandemic. Pandemic stress may have influenced the study findings, although discovery interviews were framed to prompt participants to consider themselves prepandemic. In addition, given the dependence on videoconferencing during the pandemic, community members may be more facile at using this technology now.

We found it was feasible to conduct a virtual, early-stage, community-based participatory design process with participants with limited videoconferencing experience. Initial design prototypes are currently being developed in tandem with a community-generated push notifications bank. Feedback sessions with the community design team and community members will assess our designs in preparation for clinical trial enrollment.

## Abbreviations

BCW: Behavior Change Wheel
COM-B: Capability, Opportunity, and Motivation
JITAI: just-in-time adaptive intervention
WIRED-L: Wearables In Reducing risk and Enhancing Daily Lifestyle
